# Strategies for promoting tendon-bone healing: Current status and prospects

**DOI:** 10.3389/fbioe.2023.1118468

**Published:** 2023-01-27

**Authors:** Chenhui Yang, Yuanjun Teng, Bin Geng, Hefang Xiao, Changshun Chen, Rongjin Chen, Fei Yang, Yayi Xia

**Affiliations:** ^1^ Department of Orthopedics, Lanzhou University Second Hospital, Lanzhou, China; ^2^ Orthopaedics Key Laboratory of Gansu Province, Lanzhou University Second Hospital, Lanzhou, China; ^3^ The Second School of Clinical Medical, Lanzhou University, Lanzhou, China; ^4^ Department of Orthopedic, Tianshui Hand and Foot Surgery Hospital, Tianshui, China

**Keywords:** tendon-bone insertion, anterior cruciate ligament, rotator cuff, tendon-bone healing, stem cells, cell factor, platelet-rich plasma, exosome

## Abstract

Tendon-bone insertion (TBI) injuries are common, primarily involving the rotator cuff (RC) and anterior cruciate ligament (ACL). At present, repair surgery and reconstructive surgery are the main treatments, and the main factor determining the curative effect of surgery is postoperative tendon-bone healing, which requires the stable combination of the transplanted tendon and the bone tunnel to ensure the stability of the joint. Fibrocartilage and bone formation are the main physiological processes in the bone marrow tract. Therefore, therapeutic measures conducive to these processes are likely to be applied clinically to promote tendon-bone healing. In recent years, biomaterials and compounds, stem cells, cell factors, platelet-rich plasma, exosomes, physical therapy, and other technologies have been widely used in the study of promoting tendon-bone healing. This review provides a comprehensive summary of strategies used to promote tendon-bone healing and analyses relevant preclinical and clinical studies. The potential application value of these strategies in promoting tendon-bone healing was also discussed.

## 1 Introduction

In recent years, with the extensive development of sports activities, the incidence of tendon-bone insertion (TBI) injury has increased. TBI injuries are common in rotator cuff (RC) and anterior cruciate ligament (ACL) injuries. More than 30 million TBI injuries are reported worldwide each year (Maffulli et al., 2003). Ligaments are essential for the proper movement and stability of joints. Ligament injury often leads to abnormal joint motion, secondary damage to articular cartilage and other structures, and even degenerative joint changes ((Moses et al., 2012), (von Porat et al., 2004)). Currently, the main treatment for TBI injuries is tendon/ligament repair and reconstruction, which re-establishes the physical structure of the joint through tendon grafting. In the process of tendon transplantation, tendon graft should be fixed through bone tunnel. The main factor that determines the success of surgery is the good fusion of the graft tendon with the bone tunnel. Although autologous ligament repair and reconstruction techniques have made great progress, there are still limitations. The main disadvantages of autologous tendon transplantation are the longer tendon-bone healing time and the higher recurrence rate [(Goradia et al., 2000), (Rodeo et al., 1993)].

Studies have shown that failure of tendon-bone healing can lead to failure of surgery. Scar tissue formation often occurs in tendon-bone healing, and the limited mechanical properties of scar tissue can easily lead to tendon re-injury (Galatz et al., 2004). It has been reported that the failure rate of RC prostheses is 20%–94% (Galatz et al., 2004; Xu et al., 2021), while the failure rate of ACL reconstruction is 10%–25% (Vergis and Gillquist, 1995). Additionally, tendon-bone healing can lead to complications such as knee joint relaxation, cartilage and meniscus damage, and post-traumatic osteoarthritis (Levine et al., 2013; Musahl and Karlsson, 2019). Therefore, tendon-bone healing is essential for ligament repair and reconstruction.

In order to promote tendon-bone healing, it is essential to understand the physiological process in the bone tunnel. There is evidence that the tendon-bone healing process begins with the growth of fibrous tissue cells between bone and tendon along the length of the bone tunnel, gradually forming a layer of collagen fibers. As the collagen layer matures, the trabecular bone around the tendon is reconstructed, and the tendon-bone interface increases in strength. Ultimately, the strength of the tendon-bone interface is determined by the bone’s entry, mineralization, and maturation (Rodeo et al., 1993). Effective bone ingrosion promotes tendon-bone healing and is an important factor in speeding recovery and restoring limb function. In addition to the mesenchymal stem cells (MSCs) participating in tissue repair through differentiation, this physiological processes also involve the regulation of a wide range of cellular factors.

Researchers have been developing techniques to promote bone growth during tendon transplantation for the past 10 years, and have developed a number of therapeutic strategies based on the physiological process of tendon-bone healing. Among them, biomaterials (Lacheta and Braun, 2022), platelet-rich plasma (PRP) (Figueroa et al., 2015), cell factors (Zhu et al., 2022a), stem cells (Xu et al., 2021), exosomes (Xu et al., 2022a)and physical therapy (Lai et al., 2021) and other technologies have been widely used in the basic research of promoting tendon-bone healing, and have achieved good efficacy. Some of these technologies have also been applied in clinical practice. In this review, preclinical and clinical studies are analyzed to summarize current strategies used to promote tendon-bone healing. To provide theoretical basis for clinical treatment of promoting tendon-bone healing, we discussed the potential application value of the above strategies.

## 2 Strategies to promote tendon-bone healing

**FIGURE 1:**
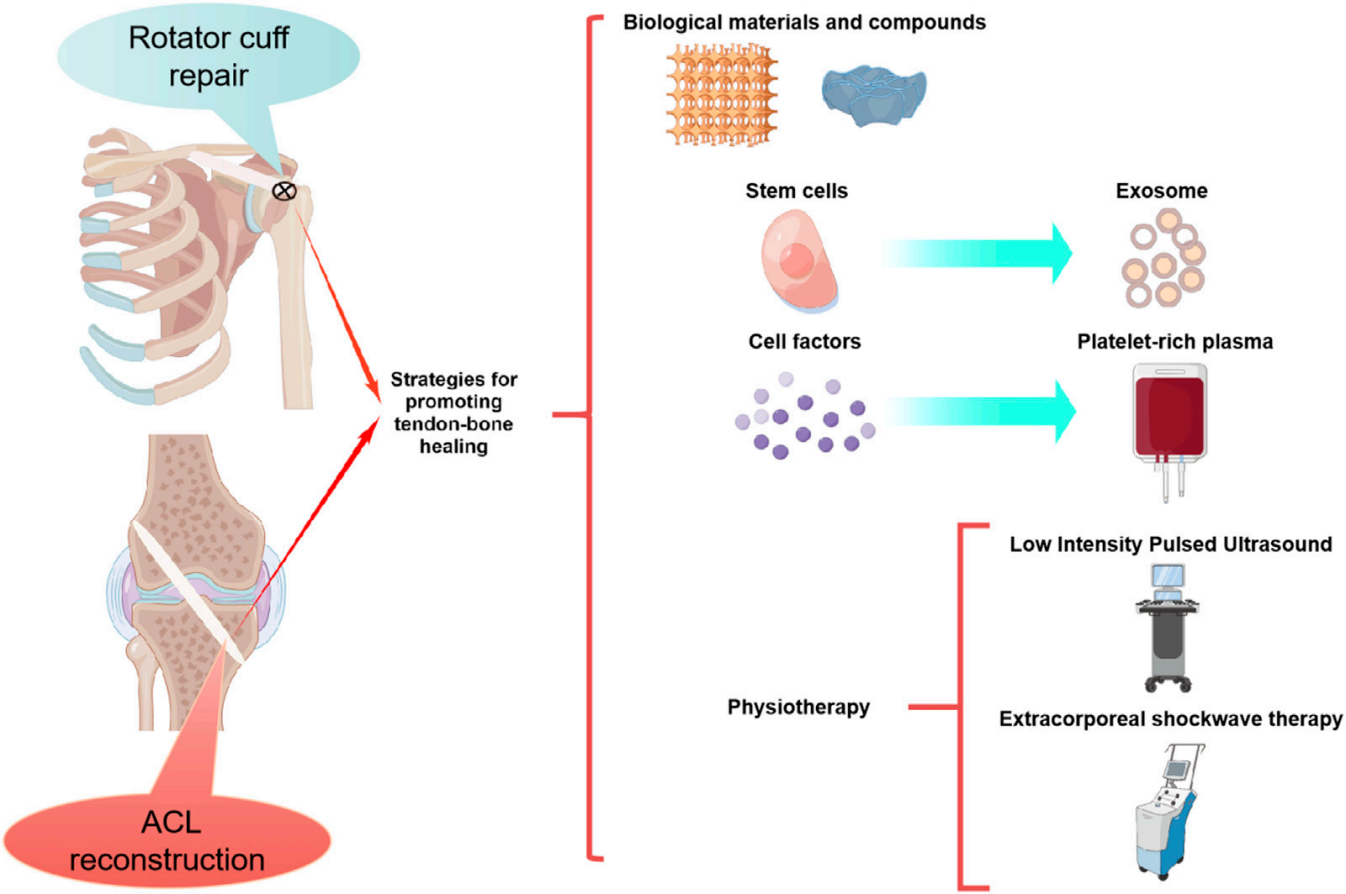
Strategies for promoting tendon-bone healing were demonstrated by Figdraw.

It is well known that the main factors determining the perfect healing of the tendon-bone interface are the formation of blood vessels and bone (Zhao et al., 2022). In recent years, researchers have directly developed stem cells, cell factor, physical and other treatments for these two factors. Since drug localization and sustained release are difficult due to the specificity of joint local administration, biomaterials and compounds have been developed for adjuvant drug therapy. Furthermore, PRP, stem cells that overexpressed target genes and marrow mesenchymal stem cell-derived exosomes (MSC-Exos) have been developed in addition to stem cell and cytokine therapy (Figure 1). Numerous preclinical studies have been conducted using the above methods, and good results have been achieved (Tables 1–8).

**TABLE 1:** Preclinical studies of biomaterials and compounds used to promote tendon-bone healing.

Intervening measure	Method of delivery	Treatment outcomes	Animal model	References
Cap	Soaking the tendon graft	The Cap hybrid tendon can promote sineoskeletal healing at the articular foramen of the anterior femoral and posterior tibial tunnels.	Goat ACL reconstruction	Mutsuzaki et al. (2016)
Cap	Soaking the tendon graft	The microstructures of Cap hybrid tendon grafts contain low crystalline apatite and type I collagen, and thus resemble the microstructures of bone, enhancing the formation of new bone in the bone tunnel.	Rats ACL reconstruction	Mutsuzaki et al. (2017)
Strontium-enriched Cap cement (Sr-CPC)	Coating the tendon graft	Sr-CPC promotes osteoblast-like cell proliferation, increases alkaline phosphatase (ALP) activity and the expression of type Ⅰ collagen, osteocalcin, and osteostatin, and accelerates healing in bone tunnels.	Rabbit ACL reconstruction	Kuang et al. (2013)
Simvastatin coupled gelatin hydrogel	Local administration	Local low-dose simvastatin coupled gelatin hydrogel promotes early tendon-bone healing through angiogenesis and osteogenesis.	Rabbit ACL reconstruction	Oka et al. (2013)
Strontium-enriched Cap cement (Sr-CPC)	Local administration	Sr-CPC accelerates graft healing in bone tunnels.	Rabbit ACL reconstruction	Kuang et al. (2014)
Demineralized bone matrix(DBM)	Local administration	Tendon-bone healing is enhanced by DBM, which promotes early differentiation of mesenchymal cells into chondrogenic and osteoblastic lineages.	Rabbit ACL reconstruction	Hsu and Wang, (2014)
Kartogenin (KGN)	Local injection	KGN induces chondrogenic differentiation of stem cells and promotes tendon-bone healing.	Model of Achilles tendon and patellar tendon injury in rabbits	Zhang and Wang, (2014)
Ca-5(PO_4_)(2)SiO_4_ (CPS) HA	Local administration	Both CPS and HA bioceramics contribute to cell attachment and proliferation, and promote new bone formation.	Rat RC repair model	Zhao et al. (2014a)
bFGF-loaded electrospun poly(PLGA) fibrous membrane	Local administration	bFGF-PLGA membrane facilitates cell attachment and proliferation, and accelerates tendon-bone reconstruction.	Rat RC repair model	Zhao et al. (2014b)
Biomimetic nanofiber membrane of polycaprolactone/nanoHA/collagen(PCL/nHAp/Col)	Wrapping graft tendon	PCL/nHAp/Col nanofiber membrane can effectively promote the healing of tendon and host bone and improve the mechanical strength during ACL reconstruction.	Rabbit ACL reconstruction	Han et al. (2015)
Microfibers of poly (3-caprolactone) (PCL) and nanofibers of chitosan	Local implanting	The PCL-chitosan scaffold enhanced new bone formation and collagen and glycosaminoglycan expression, and it also enhanced the strength of the regenerated TBI site.	Rat RC repair model	Zhao et al. (2015)
Biodegradable high-purity magnesium (HP Mg)	Local implanting	HP Mg is a biodegradable interfering screw that has the potential to promote the regeneration of fibrocartilage implants in ACL reconstruction.	Rabbit ACL reconstruction	Cheng et al. (2016)
Biodegradable polylactide bolt as the bone anchor and apoly (d,l-lactide-co-glycolide) nanofibrous membrane embedded with collagen as a biomimic patch	Local implanting	The composite polymer of nanofiber membrane can effectively promote rabbit tendon reconstruction, reduce tunnel enlargement and enhance tendon-bone fusion.	Rabbit ACL reconstruction	Chou et al. (2016)
Electrospun silk fibroin (SF) mat	Coating the tendon graft	SF mat can promote tendon-bone healing in soft tissue graft.	Rabbit ACL reconstruction	Zhi et al. (2016)
Collagen gel loaded with BMP-2	Local injection	Collagen gel loaded with BMP-2 can be used to enhance the healing of the tendon-bone interface.	Rabbit RC repair model	Lee et al. (2017)
P(2) porous titanium-coated constructs (DJO Surgical, Austin, TX, United States)	Local implanting	Osseointegration P2 porous titanium-coated prosthesis reduces superficial and deep tissue infection. Thus, tendon-bone healing can be improved with P (2) porous titanium implants.	Rat RC repair model	Tucker et al. (2017)
Mg screws	Local implanting	Mg screw has good corrosion resistance, degradation will not cause bone tunnel widening. Mg screw can promote tendon-bone healing.	Rabbit ACL reconstruction	Wang et al. (2017a)
Polyvinylpyrrolidone-iodine (PVP-I)	Soaking the tendon graft	PVP-I promotes tendon-bone healing through osteogenesis.	Rabbit achilles tendon repair model	Zhang et al. (2017a)
Dual-layer aligned-random scaffold (ARS)	Local implanting	ARS can effectively enhance the fusion of tendon and bone and promoting the maturation of collagen tissue.	Rabbit achilles tendon repair model	Cai et al. (2018)
Tendon-derived hydrogel (tHG)	Local administration	tHG can induce ADSCs to locate at the site of tendon injury to improve tendon-bone healing.	Rat tendon repair model	Franklin et al. (2020)
Chitosan/gelatin/β-glycerol phosphate(C/G/GP) hydrogels	Local administration	Collagenase C/G/GP hydrogel promotes tendon-bone healing.	Rabbit ACL reconstruction	Huang et al. (2020a)
Silk-collagen scaffold with both ends modified by HA	Local implanting	The silk-collagen scaffold promote bone fusion, which has great potential for clinical application.	Rabbit ACL reconstruction	Bi et al. (2021)
Amorphous calcium phosphate (ACP) nanoparticles	Local administration	ACP nanoparticles improve the biomechanical strength of the tendon-bone junction, promote osteogenesis and angiogenesis.	Rat RC repair model	Liao et al. (2021)
Mg-pretreated periosteum (M-P)	Coating the tendon graft	M-P significantly increased the formation of fibrocartilage, and prevented bone loss around the tunnel and more bone growth into the tendon graft.	Rabbit ACL reconstruction	Wang et al. (2021)
Fibrin Glue-Kartogenin Complex(FG-KGN)	Local administration	FG-KGN complex can effectively promote the regeneration and formation of fibrochondral tissue.	Rabbit RC repair model	Zhu et al. (2021)
HA incorporated polylactic acid (PLLA) aligned nanofibrous membranes	Coating the tendon graft	Electrospun PLLA-HA nanofiber membrane can better induce bone formation of BMSCs.	Rat RC repair model	Lv et al. (2022)
HA/type I collagen (Hap/Col I) paste	Local injection	Hap/Col I provides and microenvironment for early cell attachment and proliferation, further osteogenic expression and extracellular matrix deposition.	Canine ACL reconstruction	Jiang et al. (2023)
Natural fish scale (FS) modified by calcium silicate nanoparticles (CS NPs)	Local fixation	CS-FS promotes the repair of transitional tissue, and plays a more active integration role in promoting the repair of bone-tendon interface.	Rat and rabbit RC repair model	Han et al. (2023)

**TABLE 2:** Preclinical studies of stem cells used to promote tendon-bone healing.

Intervening measure	Method of delivery	Treatment outcomes	Animal model	References
Non-autologous transplantation of hUCB-MSCs	Local administration	hUCB-MSCs can enhance tendon-bone healing by promoting broad fibrochondrogenesis, with higher histological scores and reduced femoral and tibial tunnel widths.	Rabbit ACL reconstruction	Jang et al. (2015)
BMSCs genetically modified with BMP2 and bFGF	Local administration	Gene modified BMSCs have better structure, promote new bone formation and higher mechanical properties, and contribute to the healing process.	Rabbit ACL reconstruction	Chen et al. (2016)
ADRC	Local administration	ADRCs can be used to enhance graft healing during ACL reconstruction. Local administration facilitates the early healing process at the tendon-bone junction, both histologically and mechanically.	Rabbit ACL reconstruction	Kosaka et al. (2016)
Platelet-derived growth factor subunit B (PDGF-B) gene Modified BMSCs	Local administration	PDGF-B can increase the ultimate load and stiffness of tendon tissue, and expression of PDGF-B can enhance tendon-bone healing.	Rat RC repair model	Wang et al. (2018)
Fresh autologous bone marrow (BM)	Local administration	Local delivery of fresh autologous bone marrow was more effective than alternative therapy in promoting tendon to bone healing.	Rat autogenous tendon transplantation model	Lu et al. (2019)
hBMSCs	Local administration	The effect of hBMSCs is to accelerate graft-bone incorporation and midsubstance ligamentization as well as to enhance fibroblast proliferation, differentiation, and collagen synthesis.	Rat ACL reconstruction	Sun et al. (2019)
hBMSC	Local administration	hBMSC-CM influences macrophage polarization through the Smad2/3 signaling pathway and thus plays a role in tendon-bone healing, which is dependent on its immunomodulatory properties.	Rat RC repair model	Chen et al. (2021)
Bone marrow mononuclear cells(BMMNCs);bone marrow stromal cells (BMSCs)	Local administration	Both BMMNCs and BMSCs were equally effective at repopulating cells and healing interfacial allografts.	Rabbit ACL reconstruction	Lu et al. (2021)
ADSC sheets	Local administration	ADSC sheets can improve the biomechanical strength of the *in vivo* model, prevent bone tunnel enlargement.	Rabbit ACL reconstruction	Matsumoto et al. (2021)
BMSCs modifcated of RUNX1 with lentiviral system	Local administration	Runx1 upregulated BMSCs promote osteogenesis after ACL reconstruction by upregating the number of osteoblasts identified by ALP, osteocalcin, and osteopontin at the tendon-bone interface.	Rat ACL reconstruction	Kang et al. (2022)
De-BMSCs	Local administration	De-BMSCs transplantation can better promote bone formation at the tendon-bone interface and increase the early biomechanical strength of the reconstructed ACL.	Rabbit ACL reconstruction	Tie et al. (2022)

**TABLE 3:** Preclinical studies on the application of cell factors to promote tendon-bone healing.

Intervening measure	Method of delivery	Treatment outcomes	Animal model	References
BMP-2	Local administration	BMP-2 induces bone formation by regulating the recruitment and differentiation of bone progenitor cells, which improves tendon-bone healing and bone formation.	Rabbit ACL reconstruction	Kim et al. (2014)
CXCL13	Local administration	CXCL13 is involved in the bone regeneration process treated by MSCs through the activation of ERK1/2by miR-23a, thus promoting tendon-bone healing.	Rat achilles tendon repair model	Tian et al. (2015)
Fibrin clots loaded with bFGF and CaPP	Local administration	Fibrin clots loaded with bFGF and CaPP enhanced cell migration and proliferation, increased bioactivity at the tendon-bone interface, and promoted tendon-bone healing.	Rabbits patellar tendon repair model	Zhang et al. (2016b)
Hyaluronic acid	Local administration	Hyaluronic acid-activated MSCs may play a key role in accelerating tendon to bone healing.	Rabbit RC repair model	Honda et al. (2017)
aFGF delivered in collagen (aFGF/collagen)	Local administration	The application of aFGF/collagen can promote early healing of the tendon-bone interface, especially when using high concentrations of aFGF/collagen.	Rabbit ACL reconstruction	Lu et al. (2018a)
Collagen sponges (CS) as a delivery device for osteoprotegerin (OPG)/BMP-2	Local administration	The combination of CS and growth factor OPG/BMP-2 can ensure the slow and stable release of OPG/BMP-2 and significantly promote tendon-bone healing.	Rabbit ACL reconstruction	Wei et al. (2020)
HA encapsulated with TGF-β1	Local administration	The addition of HA-TGF-β1 ceramic powder at the tendon-bone interface can increase the formation of bone and fibrocartilage, improve collagen tissue and enhance tendon-bone healing.	Rat RC repair model	You et al. (2018)
The GF combination of TGF-β1, Insulin-like growth factor 1, and parathyroid hormone	Local administration	GF combination can promote the proliferation and differentiation of tendon cells and improve the biomechanical and histological quality of the repaired tendon-bone interface.	Rat RC repair model	Zhu et al. (2022a)

**TABLE 4:** Preclinical studies of platelet-rich application to promote tendon-bone healing.

Intervening measure	Method of delivery	Treatment outcomes	Animal model	References
PRP combined with sponge carrier	Local injection	PRP significantly improved the biomechanical properties of RC tendon—bone interface.	Rat RC repair model	Ersen et al. (2014)
PRP plus bioactive glass (BG) mixture	Local administration	PRP + BG mixture can promote tendon-bone healing in tendon repair.	Rabbit RC repair model	Wu et al. (2014)
PRP	Local injection	PRP during tendon-to-bone implantation has positive effects histologically.	Rabbits ACL reconstruction	Ağır et al. (2017)
Chitosan-PRP implants	Local implanting	Chitosan-PRP implants may modulate RC healing in large animal models.	Sheep RC repair model	Deprés-Tremblay et al. (2018)
PRP Combined with Gelatin Sponge (GS)	Local implanting	PRP-GS can promote early healing of the tendon—bone junction.	Rabbit ACL reconstruction	Zhang et al. (2019a)
Combination of graphene oxide and PRP	Local implanting	GO/PRP improved the biomechanical properties of newborn tendons and made TBI tissue more stable.	Rabbit RC repair model	Bao et al. (2021)
Bone marrow-derived PRF (BM-PRF)	Local implanting	BM-PRF can enhance tendon-bone continuity and achieve better tendon-bone maturity.	Rabbits RC repair model	Uno et al. (2022)
PRF	Local administration	By promoting the formation of fibrocartilage and bone in a transitional zone between bone and tendon graft, bioactive PRF effectively promotes bone and tendon healing.	Rabbit ACL reconstruction	Wong et al. (2020)

**TABLE 5:** Preclinical studies of exosomes used to promote tendon-bone healing.

Intervening measure	Method of delivery	Treatment outcomes	Animal model	References
BMSC-Exos	Local administration	BMSC-Exos promotes tendon-bone healing after RC reconstruction by promoting angiogenesis and inhibiting inflammation.	Rat RC repair model	Huang et al. (2020b))
BMSC-Exos	Local administration	Local administration of BMSC-Exos promotes fibrochondrogenesis by increasing M2 macrophage polarization, thereby improving biomechanical properties.	Mouse achilles tendon repair model	Shi et al. (2020)
Mesenchymal stem cell -derived exosomes(MSC-Exos)	Local injection	MSC-Exos can significantly reduce osteoclast formation and improve the strength of tendon-bone healing, and can be used as a potential therapy for osteolysis.	Mouse achilles tendon repair model	Feng et al. (2021)
Purified exosome product (PEP)	Local injection	PEP promotes the migration and fusion of osteoblasts and tendon cells, especially in direct cell-cell contact, and accelerates tendon-bone healing for RC.	Rat RC repair model	Ren et al. (2021)
Exosomes of polyaspartic acid-polylactic acid-glycolic acid copolymer (PASP-PLGA) microcapsules	Local administration	Protein expression related to tendon regeneration and cartilage differentiation was significantly up-regulated after PASP-PLGA microcapsule exosome treatment, promoting tendon-bone interface healing after RC injury.	Rabbit RC repair model	Han et al. (2022)
Exosome derived from magnetically actuated (iron oxide nanoparticles (IONPs)	Local administration	IONP-Exos significantly prevented bone loss around the tunnel, promoted more bone inward growth in the tendon graft, and increased fibrochondrogenesis at the tendon-bone tunnel interface.	Rat ACL reconstruction	Wu et al. (2022)
BMSC-Exos	Local administration	BMSC-Exos promotes the polarization of M1 macrophages to M2 macrophages through miR-23a-3p, reduces the early inflammatory response at the tendon-bone interface, and promotes the early postoperative healing.	Rat ACL reconstruction	Li et al. (2022)
Exosomes from infrapatellar fat pad (IPFP) MSCs	Local administration	IPFP MSC-derived exosomes accelerate tendon-bone healing and intraarticular graft reconstruction, which may be related to immune regulation of macrophage polarization.	Rat ACL reconstruction	Xu et al. (2022a)

**TABLE 6:** Preclinical studies of physical therapy used to promote tendon-bone healing.

Intervening measure	Method of delivery	Treatment outcomes	Animal model	References
LIPUS	Local treatment	LIPUS alters the differentiation pathway of multipotent mesenchymal cells into osteoblasts and/or chondroblasts, stimulates osteogenic differentiation of osteoblasts, and significantly increases the expression of BMP, which is involved in and improves tendon-bone healing.	Sheep RC repair model	Lovric et al. (2013)
ESW	Local treatment	ESW enhances fibrocartilage regeneration at the TBI healing interface by upregating the expression of fibrochondroscope-related manufacturers and cell factors through mechanical signaling.	Rabbit patellar tendon repair model	Chow et al. (2014)
LIPUS	Local treatment	The effect of LIPUS on tendon-bone healing from the 7th day after surgery was more significant	Rabbit patellar tendon repair model	Lu et al. (2016a)
LIPUS	Local treatment	LIPUS treatment starting at the second week after surgery can accelerate bone formation during the healing process of tendon-bone junction and significantly improve the healing quality of BTJ injury.	Rabbit patellar tendon repair model	Lu et al. (2016b)
Intermittent negative pressure	Local treatment	Intermittent negative pressure in the tendon to bone healing and creep replacement plays a positive role in rabbit ACL reconstruction.	Rabbits ACL reconstruction	Sun et al. (2017)
LIPUS	Local treatment	The healing effect of LIPUS stimulation twice a day is better than that of the treatment once a day.	Rabbit patellar tendon repair model	Lu et al. (2018b)
Pulsed electromagnetic field (PEMF)	Local treatment	PEMF downregulates genes associated with fibrotic healing responses and significantly increases the rate of osteodynamic formation and the number of osteoblasts in the humeral head directly adjacent to the tendon.	Rats RC repair model	Huegel et al. (2022)
LIPUS	Local treatment	LIPUS accelerates the accumulation of early inflammatory macrophages and promotes the polarization of late anti-inflammatory macrophages (M2). LIPUS showed a higher bone volume/total volume ratio and a better TBI maturity score after treatment.	Rat RC repair model	Xu et al. (2022b)

**TABLE 7:** Preclinical studies of combined application in promoting tendon-bone healing.

Intervening measure	Method of delivery	Treatment outcomes	Animal model	References
PRP combined with BMSCs	Local administration	The combination of PRP and BSCs promoted more maturation of the tendon-bone interface, and more newly formed bone was found in the bone tunnel wall.	Rabbit ACL reconstruction	Teng et al. (2016)
Cortical demineralised bone matrix used with minimally manipulated MSCs	Local administration	The use of DBM with minimally treated MSCs promotes healing at the sineon-bone interface with superior mechanical and histological results.	Ovine patellar tendon repair model	Thangarajah et al. (2016)
ADSCs, PRP, and Extracellular Matrix Hydrogel	Local administration	Combined application showed greater repair capacity and biocompatibility.	Rat achilles tendon repair model	McGoldrick et al. (2017)
Kartogenin (KGN) and PRP	Local administration	KGN-PRP can induce the formation of fibrocartilage.	Rat achilles tendon repair model	Zhang et al. (2017b)
Kartogenin (KGN) with PRP	Local administration	KGN with PRP as the carrier promoted the formation of fibrochondral band between tendon graft and bone interface.	Rat achilles tendon repair model	Zhou et al. (2017)
Stromal cell-derived factor 1 (SDF-1)-releasing collagen-silk (CSF) scaffold combined with intra-articular injection of LSPCs	Local administration	This treatment strategy also improved cartilage degeneration and reduced the severity of joint fibrosis.	Rabbit ACL reconstruction	Hu et al. (2018)
DBM and BMSCs	Local administration	MSCs-enhanced DBM improved RC healing after 6 weeks and brought bone density in the RC back to pre-injury levels.	Rat RC repair model	Thangarajah et al. (2018)
BMP-12-overexpressing MSCs loaded 3D-printed PLGA scaffolds	Local administration	The 3D-printed PLGA scaffold supported by BMSCs overexpressing BMP-12 can promote the healing of the tendon—bone interface, improve collagen tissue and increase fibrocartilage in rabbit trochanteric sleeve repair.	Rabbit RC repair model	Chen et al. (2019)
MSCs and PRP	Local administration	Combination therapy induced the strongest signals associated with angiogenesis, bone formation, and *in situ* tendon formation.	Rat RC repair model	Han et al. (2019a)
BMP-2 and PRF	Local administration	The combination of bone morphogenetic protein 2 (BMP-2) and PRF (PRF) therapy has a synergistic effect on sineotone-bone healing and has great potential in the treatment of ACL reconstruction.	Rat ACL reconstruction	Han et al. (2019b)
Simvastatin and PRP	Local administration	Simvastatin combined with PRP can induce chondrogenesis of BMSCs and promote fibrochondrogenesis *in vivo*.	Rat achilles tendon repair model	Zhang et al. (2019c)
Poly lactic-co-glycolic acid (PLGA) scaffolds loaded with BMSCs	Local implanting	The implantation of BMSCs-PLGA scaffolds promoted collagen formation, increased collagen diameter at the tendon-bone interface, and improved biomechanical properties of the regenerated tendons.	Rabbit RC repair model	Chen et al. (2020)
Hierarchically demineralized cortical bone coated with stem cell-derived extracellular matrix (hDCB-ECM)	Local administration	hDCB-ECM promotes the formation of bone and fibrocartilage at the tendon-bone interface.	Rabbit RC repair model	He et al. (2021)

**TABLE 8:** Clinical studies on the promotion of tendon-bone healing.

Conditions	Intervening measure	Patients (Number)	Treatment outcomes	References
ACL reconstruction	ESWT	26	ESWT significantly improves the subjective Lysholm score and decreases the middle 1/3 tibia tunnel enlargement after single hamstring autograft ACL reconstruction.	Wang et al. (2014)
RCTs	PRP	52	The application of moderately concentrated PRP improves the clinical and structural prognosis of large cuff tears. PRP can also enhance the ability of vascular repair around the injured site early on.	Pandey et al. (2016)
ACL reconstruction	ADRCs	20	ADRCs can significantly improve patient knee function and graft healing/maturation. However, this improvement wasn’t statistically significant compared to a control group undergoing ACL reconstruction alone.	Alentorn-Geli et al. (2019)
ACL reconstruction	Cap hybridization method for graft tendon	8	Cap hybridization is a safe and feasible method for ACL reconstruction. In addition, the method can improve clinical efficacy.	Mutsuzaki et al. (2019)
ACL reconstruction	Allogeneic hUCB-MSCs	10	Allogeneic hUCB-MSCs were safely used for ACL reconstruction without treatment-related adverse events. However, studies haven’t shown any evidence of clinical advantage.	Moon et al. (2021)
ACL reconstruction	PRP	85	PRP can promote graft tendon-bone healing and improve early postoperative status and joint function.	Chen et al. (2022)
ACL reconstruction	PRP	30	PRP had no significant effect on promoting tendon-bone healing and improving knee function. However, PRP may promote intraarticular graft maturation.	Gong et al. (2022)

### 2.1 Biological materials and compounds

With the rapid development of bioengineering technology over the past few years, the research of biomaterials and compounds for promoting tendine-bone healing has also made rapid progress. Biological products such as Calcium phosphate (Cap), Biological nanofiber scaffolds, magnesian products and hydrogel have been widely used in preclinical studies and have achieved good results (Table 1).

Cap material has the advantage of promoting bone formation and has been widely used to enhance bone regeneration. In order to explore the effect of Cap on tendon-bone healing, MUTSUZAKI H et al. (Mutsuzaki et al., 2016; Mutsuzaki et al., 2017) used Cap to soak tendon grafts to try to improve tendon-bone healing. In the treatment of the goat ACL reconstruction model, Cap hybrid tendon grafts showed significant histological improvement in both bone tunnels and increased *in-situ* force under tibial preload applied at high flexion angles. This research suggests that this technique may promote tendon-bone healing by enhancing osteogenesis. Liao et al. (2021) recently studied the application of amorphous Cap nanoparticles in tendon-bone healing, and found that amorphous Cap nanoparticles couldn’t only improve the biomechanical strength of the tendon-bone junction by promoting osteogenesis, but also promote angiogenesis, thus promoting tendon-bone healing. Secondly, the effects of Ca-5(PO_4_) (Moses et al., 2012) SiO_4_ (CPS) and hydroxyapatite (HA) on promoting tendon-bone healing were compared. Zhao et al. (2014a) found that the local application of CPS and HA bioceramics both contributed to cell attachment and proliferation, and promoted the formation of new bone. Compared with HA, CPS bioceramics have a more significant effect on tendon-bone healing, which is expected to improve healing after RC injury repair. Strontium (Sr) has good bone conductivity and is widely used as a bone substitute to treat bone diseases. Strontium-enriched calcium phosphate cement (Sr-CPC) was developed by researchers (Kuang et al., 2013). It is found that Sr-CPC has the potential to enhance the fusion of graft and bone tunnel in ACL reconstruction. Preclinical studies have shown that Sr-CPC promotes osteoblast-like cell proliferation, and increases alkaline phosphatase (ALP) activity and the expression of type I collagen, osteocalcin, and osteoprotetin (Panzavolta et al., 2008; Kuang et al., 2012).

In recent years, researchers have attempted to mimic the tendon-bone healing interface through tissue engineering. However, because the tendon-bone healing interface is a transition of soft to hard tissue, the structure is complex. The interfacial connection of tendon-bone healing was observed in different animal models, and it was found that the interfacial connection was mainly composed of Sharpey-like fibers (Tabuchi et al., 2012; Kawakami et al., 2017). Based on this structural property, biological nanofiber scaffolds prepared by electrospinning technology have been developed and widely used in tissue repair of tendon-bone interface (Zhao et al., 2014b; Han et al., 2015). Biological nanofiber scaffolds have the characteristics of good biocompatibility, high aspect ratio and high porosity (Chen et al., 2009; Zhang et al., 2010). In addition, the morphological characteristics of scaffolds play an important role in the adhesion, proliferation and differentiation of active cells (Huang et al., 2016; Mahmoodinia Maymand et al., 2018). Yin et al. (2010) studied the effect of electrospinning nanoscaffolds on the differentiation of human tendon stem/progenitor cells (hTSPCs), and the results showed that nanoscaffolds could induce the tenogenic and osteogenic differentiation of hTSPCs. Cai et al. (2018) prepared a double-layered aligned-random scaffold (ARS) by electrostatic spinning. In the experiment, ARS was used to wrap the Achilles tendon and transplant it through the bone tunnel. The results showed that ARS could effectively enhance the tendon-bone fusion by increasing the area of fibrocartilage and inducing the formation of new bone. Similarly, researchers have experimented with nanoscaffolds loaded with growth factors to promote tendon-bone healing Zhao et al. (2014b) invented an electrospun polylact-glycol ester (PLGE) fiber membrane loaded with bFGF for repairing rotator cuff tear (RCT). The results showed that electrospinning fiber membrane was conducive to cell attachment and proliferation, and accelerated sineoskeletal reconstruction. PLGE fiber membrane loaded with bFGF had a more obvious effect on sineoskeletal healing. In addition, Bi et al. (2021) prepared silk collagen scaffolds loaded with HA and used HA/sericinogen scaffolds as grafts to replace the original ACL in animal models, and the histological staining after surgery showed that a large number of mature bones were formed at the stens-bone interface. Immunohistochemical staining showed more deposition of type I collagen and osteocalcin than that of the control group. These results indicate that the direct use or drug loading of nanoengineered biological scaffolds is a promising method to promote tendon-bone healing.

Magnesium is a biodegradable biomaterial with good biocompatibility and mechanical properties, which can meet the load requirements during knee implantation and active rehabilitation (Farraro et al., 2014). Studies have shown that Mg ion can promote the growth of transplanted tendon into bone through the osteogenic differentiation pathway of stem cells (Yoshizawa et al., 2014). (Cheng et al., 2016) reported the efficacy of biodegradable high-purity magnesium (HP Mg) screw in promoting tendon-bone healing after ACL reconstruction. Research results showed that HP Mg screw can promote the expression of BMP-2, VEGF and glycosaminoglycan in the early stage of tendon-bone healing. Based on the results of this study, magnesium ions are responsible for promoting fibroblast cartilage regeneration by acting on the above molecules. Wang et al. (2017a) compared the efficacy of titanium screw and magnesium screw in promoting tendon-bone healing. The results showed that Mg screw could significantly improve the healing quality of bone marrow tract by promoting the ossification of tendon endothelial area. It was also found that Mg screw can significantly promote the bone formation in the periscrew area at the early stage of healing, and has good corrosion resistance and doesn’t cause bone tunnel enlargement after degradation. Therefore, Mg screw can be used instead of titanium screw in ACL reconstruction. In addition, Wang J et al. found that the release of calcitonin gene-related peptide played a significant role in promoting osteogenic differentiation by activating the cyclic adenosine monophosphate (cAMP) reaction element binding protein pathway, and this effect was significantly enhanced with the increase of magnesium ion concentration in periosteum (Zhang et al., 2016a; Wang et al., 2020). It has also been demonstrated that a higher magnesium ion concentration enhances cell adhesion strength by activating the integrin/local adhesion kinase pathway (Wang et al., 2017b). Cell migration and adhesion are mediated by periosteum proteins through the integrin/FAK pathway. (Qin et al., 2015). Therefore, Mg ions may also affect the secretion of periosteum-derived stem cells (PDSCs). Recent studies have shown that periostein can control bone regeneration potential by up-regulating PDSCs to promote osteogenic differentiation (Duchamp de Lageneste et al., 2018). Wang et al. (2021) used Mg pretreated periosteum wrapped graft tendon, and found that tendon-bone healing was significantly enhanced. These studies suggest that the mechanism of promoting bone growth through Mg ions may be a practical therapeutic strategy to enhance the healing of the tendon-bone interface.

Currently, local injection of bone cement, growth factors and cell transplantation have been identified as potential therapeutic options for promoting tendon-bone healing (Fu et al., 2014). However, how to deliver the drug at the optimal concentration and speed in the tendon-bone healing area remains one of the clinical challenges. Hydrogel microspheres have the advantages of prolonged drug retention time and high drug loading efficiency, and have become a potential excellent drug carrier in the biomedical field. Franklin et al. (2020) demonstrated that hydrogel alone could also promote tendon-bone healing by inducing ADSCs to gather at the tendon-bone interface. Oka et al. (2013) implanted simvastatin coupled gelatin hydrogel into the bone tunnel reconstructed by ACL. It has been found that simvastatin coupled gelatin hydrogel can promote tendon-bone healing through angiogenesis and osteogenesis in the early stage. Similarly, (Lee et al., 2017) confirmed that collagen gel could be used as an effective carrier of BMP-2 to enhance the healing of the tendon-bone interface. Other researchers have combined hydrogels with collagenase for tendon-bone healing. In addition, (Huang et al., 2020a) studied and prepared chitosan/gelatin/β-glycerophosphoric acid (C/G/GP) hydrogel, which was used in combination with collagenase carrier at the tendon-bone junction. The results showed a significant increase in total bone volume in the model treated with collagenase and hydrogel. The above studies indicate that hydrogel alone or as a drug carrier can promote tendon-bone healing, so hydrogel technology may be a promising avenue for the treatment of tendon injuries in the future.

### 2.2 Stem cells

In addition to self-regeneration, Bone marrow mesenchymal stem cells (BMSCs) can differentiate into a variety of cell types, and are widely used in tissue repair and regeneration (Xu et al., 2021). Conventional BMSCs were collected from the pelvis, femur, tibia, and humerus. Some recent studies have shown that BMSCs can be obtained from synovial and adipose tissues, so these tissues have been widely used in tendon-bone repair (De Bari et al., 2001; Hanahan and Weinberg, 2011). Stem cells from different sources have different differentiation ability, and the direction of stem cell differentiation is also regulated by cell factors. Therefore, with the application of combined cytokine and gene overexpression strategies, various types of stem cells have the ability to improve tendon-bone healing (Table 2).

Human umbilical cord blood MSCs (hUCB-MSCs) are used in the study of promoting tendon-bone healing. Jang et al. (2015) studied the safety of non-autologous hUCB-MSCs in animal models of ACL reconstruction and the efficacy of promoting tendon-bone healing. This study found that non-autologous hUCB-MSCs transplantation could be used for ACL reconstruction without early immune rejection. Secondly, more broad fibrochondrocytes were formed and histological scores were higher during tendon-bone healing, and the bone tunnel width was significantly reduced. This study confirms the therapeutic potential of non-autologous hUCB-MSCs in promoting tendon to bone healing. However, it is difficult for hUCB-MSCs to be widely carried out due to ethical factors. Recently, (Tie et al., 2022) compared the efficacy of dedifferentiated osteoblast BMSCs (De-BMSCs) and BMSCs in ACL reconstruction. In this study, De-BMSCs were shown to increase the early biomechanical strength of ACL reconstructions by stimulating bone formation at the tendon-bone interface. The results of this study suggest that De-BMSC transplantation might be more effective for bone formation in the tunnel during tendon repair and reconstruction.

With the increase of stem cell sources, adipose-derived stem cells (ADSCs) have been gradually applied in the study of tendon repair. It has been demonstrated in rabbit ACL reconstruction models that ADSCs can enhance tendon-bone healing (Kosaka et al., 2016) Both histologically and mechanically, local administration promoted the early healing process of the tendon-bone junction. Similarly, (Matsumoto et al., 2021) studied the effect of ADSCs grafts on the biomechanical strength of the tendon-bone interface after ACL reconstruction. The results displayed that ADSCs tablets could enhance the biomechanical intensity of rabbit model and prevent the enlargement of bone tunnel. However, (Kaizawa et al., 2019) found that hydrogel loaded with ADRCs didn’t significantly improve tendon-bone healing after RC repair in a chronic RCT model. Therefore, more studies are needed to examine the efficacy of ADRCs in promoting tendon-bone healing after RCT repair.

Furthermore, significant progress has been made in the identification of clonability, pluripotency and self-renewal ability of tendon-derive stem cell (TSCs) in humans and mice (Bi et al., 2007). Studies have shown that TSCs have stronger clonability than BMSCs (Tan et al., 2012). These cells not only have universal stem cell properties similar to BMSCs, but also feature high expression of genes and proteins associated with tendons (Bi et al., 2007). In recent years, researchers have also explored the effectiveness of TSCs in the repair of tendon-bone injury. Shen et al. (2012) investigated the effectiveness of knitted silky collagen sponge scaffolds seeded with TSCs. After operation, the growth of fibroblasts increased and lymphocyte infiltration decreased at the implantation site of TSCs allogeneic scaffolds. Moreover, the structural and biomechanical properties of the allogeneic TSCs transplantation group were improved. This study demonstrated that the woven silk-collagen sponge scaffolds of allogeneic TPCs improve RC tendon regeneration by differentiating into tendon cells and secreting anti-inflammatory cytokines. Although MSCs are commonly used for tendon-bone junction repair isolated from various tissues, the use of TSCs to repair tendon-bone junctions may be beneficial because the tendon environment is ideal and familiar, facilitating transplantation and differentiation between transplanted cells (Lui and Wong, 2012). The research of TSCs in the treatment of tendon-bone junction injuries is still in its infancy. Further studies are needed to test the efficacy of tspc application in large animal models and to determine its efficacy *in vivo*.

Researchers have also investigated the effect of human bone marrow stem cells (hBMSCs) on tendon-bone healing. Sun et al. (2019) explored the effect of hBMSCs on tendon-bone fusion in a rat model. The results demonstrated that hBMSCs could accelerate tendon-bone fusion by enhancing fibroblast proliferation, differentiation and collagen synthesis. Similarly, (Chen et al., 2021) examined the effect of hBMSCs on promoting tendon-bone healing in RC repair models. The results of this study show that hBMSCs are capable of promoting tendon-bone healing following RC injuries in rats. Further studies confirmed that the benefits of hBMSCs on tendon-bone healing are related to the regulation of macrophages, and hBMSCs affect the polarization of macrophages through the Smad2/3 signaling pathway. In addition, (Lu et al., 2021) compared Bone marrow mononuclear cells (BMMNCs) and bone marrow stromal cells (BMSC) to promote tenon-bone healing in ACL reconstruction models. In this study, BMMNCs and BMSC were found to be equally effective in promoting tendon-bone healing. These studies suggest that the collection of hBMSCs or mononuclear cells from peripheral blood may also be a potential therapeutic option for promoting tendon-bone healing.

The differentiation ability of BMSCs varies due to different sources. Therefore, the researchers used genetic engineering to modify MSCs to target differentiation to promote tendon-bone healing. Chen et al. (2016) evaluated the effects of BMP2 and bFGF gene modified BMSCs on tendon-bone healing. This study confirmed that BMSCs genetically modified with BMP2 or bFGF can better promote new bone formation, resulting in higher mechanical properties of bone tissue, and thus more effectively promote tendon-bone healing. The study also found that the combination of overexpression of the two genes was more effective than single overexpression therapy. Similarly, (Wang et al., 2018) modified BMSCs by platelet-derived growth factor subunit B (PDGF-B) on tendon-bone healing after RCT repair. This study also found that BMSCs overexpressing PDGF-B could enhance tendon-bone healing after RCT repair. In addition, a recent study by (Kang et al., 2022) found that RUNt-related transcription factor 1 (RUNX1) gene modified MSCs also produced better efficacy in tendon-bone healing. The above studies indicate that genetic engineering technology can make up for the lack of differentiated ability of BMSCs from different sources.

### 2.3 Cell factors

A growing body of research has focused on promoting tendon-bone healing within bone tunnels through the application of cell factors, such as transforming growth factor (TGF), bone morphogenetic protein (BMP), fibroblast factor (FGF), CXCL13, Hyaluronic acid, etc., (Tian et al., 2015; Honda et al., 2017; Lu et al., 2018a; You et al., 2018; Wei et al., 2020). Local application of cell factors has been studied to promote tendon-bone healing and has shown good efficacy in animal models of tendon repair (Table 3).

Transforming growth factor β (TGF-β) family exerts a crucial function in connective tissue development (Kim et al., 2011). TGFβ1 has been shown to promote the formation of fibrous tissue at the site of tendon healing by inhibiting the matrix metalloproteinase pathway (Arimura et al., 2017), and has also been reported to effectively enhance the biomechanical properties of tendons or ligaments (Anaguchi et al., 2005). You et al. (2018) tried to apply TGFβ1-coated HA to repair RCTs, and found that it could significantly enhance the healing of the tendon-bone interface. Further studies showed that the addition of HA-TGFβ1 ceramic powder at the tendon-bone interface could increase the formation of bone and fibrocartilage and improve the collagen structure. Recently, (Zhu et al., 2022a) explored the efficacy of the combination of TGFβ1, insulin-like growth factor 1 and parathyroid hormone in improving the repaired tendon-bone interface. This study found that the combination of cytokine therapy significantly improved the quality of tendon-bone healing and the formation of a mature tendon-bone interface. These studies suggest that TGFβ1 alone or in combination can improve tendon-bone healing, providing a theoretical basis for further clinical application.

BMP-2, a widely distributed glycoprotein in bone matrix, can induce bone formation *in vivo* by regulating the recruitment and differentiation of bone progenitor cells (Bessa et al., 2008). Tendon-bone healing has been demonstrated to be enhanced by BMP-2 by increasing the biomechanical strength between the two (Ma et al., 2007). Due to its short half-life and structural instability, BMP-2 requires high doses, resulting in adverse effects (Carragee et al., 2011). KIM J G et al.^[82]^ evaluated the effect of local application of BMP-2 on bone formation and tendon-bone healing. The results showed that BMP-2 alone could promote new bone formation and bone maturation at the tendon-bone interface, thereby improving the biomechanical strength at the tendon-bone interface. In addition, Huang et al. used Collagen sponge ES (CS) combined with BMP-2 to repair bone defects in rats, and the results showed that using CS as a carrier could effectively promote tendon-bone healing (Huang et al., 2015). Similarly, (Wei et al., 2020) studied the effect of CS as a delivery device of OPG/BMP-2 on tendon-bone healing. It was found that the combination of CS and growth factor OPG/BMP-2 can induce the slow and stable release of OPG/BMP-2, and significantly promote the tendon-bone healing in the ACL model.

As members of the FGF family, both acid fibroblast growth factor (aFGF) and bFGF significantly promoted the mitosis of osteoblasts and chondrocytes, and stimulated the formation of new capillaries (Ide et al., 2009; de Girolamo et al., 2015). Therefore, researchers began to try to use aFGF and bFGF to improve tendon-bone healing. Lu et al. (2018a) applied collagen containing different concentrations of aFGF to the tendon-bone interface to evaluate the influence of aFGF/collagen on the healing of the sineoskeletal interface. According to the results, the application of aFGF/collagen composite could promote the early healing of the tendon-bone interface through the growth of new bone, especially the application of high concentration of aFGF/collagen. In addition, (Ide et al., 2009) also demonstrated that local tendon repair with bFGF-treated tendons showed early improvements in bone growth and biomechanical strength at the tendon-bone interface. The results of these studies suggest that FGF may enhance tendon-bone healing, particularly in the early stages after reconstruction.

In the process of cell migration, CXC chemokine ligand 13 (CXCL13) plays a significant role (Zhou et al., 2013). The inflammatory cytokine IL-6 significantly induces CXCL13 expression in human osteoblasts, while CXCL13 expression is not obvious in osteoclasts (Singh et al., 2009), suggesting that CXCL13 may play a role in tendon-bone healing. Tian et al. (2015) evaluated the effect of CXCL13 on tendon-bone healing in rats. The results demonstrated that CXCL13 acted on MSCs through the activation of ERK1/2 signaling pathway by miR-23a, thus promoting tendon-bone healing. Furthermore, hyaluronic acid belongs to the glycosaminoglycan family and is usually found in the synovial fluid of joints, cartilage and other tissues. There is evidence that HA plays an important role in repairing tissue damage (Neuman et al., 2015). Honda et al. (2017) explored the effect of hyaluronic acid on tendon-bone healing after RCT repair. It was found that hyaluronic acidon accelerated tendon-bone healing, enhanced biomechanical strength, and increased chondroid formation and tendon maturation at the tendon-bone interface in tendon repair models. These studies suggest that both CXCL13 and hyaluronic acidon can promote tendon-bone healing by activating the MSCs pathway, and they are also potential candidate cell factors for promoting tendon-bone healing.

### 2.4 Platelet-rich plasma

Because many cell factors are involved in tendon repair, cell factors are widely used in tendon repair. PRP, as a source of cell factors, has been shown to improve inflammation, promote cell proliferation, and enhance angiogenesis (Lang et al., 2018; Santos et al., 2018). PRP has also been identified as a potential therapy for accelerating tendon repair. Studies on the application of PRP in the repair of soft tissue wounds have confirmed its efficacy in promoting the healing of tissue wounds (Table 4). PRP is involved in tissue remodeling, chondrogenic differentiation, and wound healing by releasing cell factors (Mehrabani et al., 2019).

There have been numerous studies investigating the efficacy of PRP in reconstructing the ACL and repairing the RCT. Ağır et al. (2017) evaluated the effect of PRP on tendon-bone healing in ACL reconstruction. The results demonstrated that the use of PRP during tendon implantation into the bone tunnel could promote tendon-bone healing by inducing fibrochondral formation early on. Besides, in order to optimize the release of PRP in the joint, the role of PRP combined with drug carriers in promoting tendon-bone healing was also explored.

Zhang et al. (2019a) explored the effect of PRP combined with gelatin sponge (GS) on tendon-bone healing. It has been shown *in vitro* that GS-loaded PRP can prolong its bioactivity time and promote BMSC proliferation. PRP promotes early healing of tendon-bone junctions in a model of ACL reconstruction. However, (Ersen et al., 2014) previously found that PRP improved the biomechanical properties of RC tendon-bone interface regardless of whether GS was applied. The different models used in the two studies may account for the different results. PRP in combination with other biomaterials has also been studied to promote tendon-bone healing. Graphene oxide (GO) can promote tissue repair by improving the physical properties of tissues. Bao et al. (2021) prepared PRP gel containing different concentrations of GO to promote tendon-bone healing in rabbit tendon reconstruction model after RC injury. The results demonstrated that the addition of GO enhanced the biomechanical properties of the newly formed tendons and made the tendon-bone interface more stable. Therefore, the combination of PRP and GO has great potential in the treatment of RCT. In addition, platelet-rich fibrin (PRF), as a concentrated product of PRP, has also been used in the study of promoting tendon-bone healing. A recent study by (Uno et al., 2022) found that PRF significantly enhanced tendon-bone continuity in the RCT model, and at the same time achieved better tendon-bone maturation. Consequently, PRP and its concentrate have great potential to promote tendon-bone healing.

### 2.5 Exosome

In recent years, more and more evidence shows that BMSCs are effective in the treatment of joint diseases (Zhang et al., 2019b). BMSCs are involved in many regulatory functions through the paracrine capacity of exosomes (Kourembanas, 2015). Exosomes are bilayer extracellular vesicles containing small RNAs and proteins and are considered to be important mediators for genetic exchange and communication between cells. Recently, researchers have found that stem cell-derived exosomes can improve the healing rate of tendon repair (Zhang et al., 2021). Therefore, many studies have been carried out on exosomes in promoting tendon-bone healing, providing more theoretical basis for promoting tendon-bone healing (Table 5).

The number of studies on exosomes that promote tendon-bone healing has increased significantly in recent years. Huang et al. (2020b) Xu et al. (2022a) explored the role of bone marrow mesenchymal stem cell-derived exosomes (BMSC-Exos) in tendon-bone healing after RC and ACL reconstruction respectively. The results confirmed that BMSC-Exos can increase angiogenesis and bone growth at the tendon-bone interface, which may be related to immune regulation of macrophage polarization. Further studies by (Shi et al., 2020) found that local administration of BMSC-Exos promoted the formation of fibrocartilage by mediating the polarization of M2 macrophages, thus improving the biomechanical properties of the tendon-bone interface. In addition, LI Z et al.^[117]^ observed the effect of BMSCs-Exos on ACL reconstruction posterior tendon bone healing and its possible mechanism *in vitro* and *in vivo*. According to the results, BMSC-Exos promoted the polarization of M1 macrophages to M2 macrophages through miR-23a-3p, reduced the early inflammatory response at the tendon-bone interface, and promoted early healing after ACL reconstruction. BMSC-Exos could be used in the repair of tendons and bones based on the findings of this study. In conclusion, BMSCs-Exos has been shown to be an effective and viable natural drug for enhancing tendon-bone healing, suggesting that BMSCs-Exos may be a promising therapeutic strategy.

### 2.6 Physiotherapy

In recent years, non-invasive physical therapy has been gradually applied to promote tendon-bone healing. Low intensity pulsed ultrasound (LIPUS), as a non-invasive physiotherapy technique, has been shown to promote recovery from musculoskeletal injuries. LIPUS transmits high frequency acoustic pressure waves and mechanical stresses through the skin to biological tissues. It has been successfully demonstrated in animal models and clinical trials to stimulate bone growth and thus promote fracture healing (Hannemann et al., 2014). In addition, it has been shown that extracorporeal shock wave (ESW) can promote tendon-bone healing by upregating the expression of fibrochondrogenic cell factors through mechanical signaling. LIPUS and ESW, as non-invasive physical therapy, have also been studied in the field of promoting tendon-bone healing, and have achieved good efficacy (Kuo et al., 2007; Greve et al., 2009) (Table 6).

In 2013, Lovric et al. (2013) conducted the first study to test the effect of LIPUS on the initial tendon-bone healing in RC sheep model. The results suggest that LIPUS may help RC repair in the initial stages of tendon-bone healing. Afterwards, LU H’s team conducted a large number of studies in this field (Lu et al., 2016a; Lu et al., 2016b; Lu et al., 2018b). First, they determined the optimal time for LIPUS treatment, and the study found that LIPUS treatment on the 7th day after surgery had a more significant impact on tendon-bone healing. Further studies showed that LIPUS accelerated bone formation during tendon-bone healing from the second week after treatment, significantly improving the quality of tendon-bone healing. Finally, they evaluated the dose-effect of LIPUS stimulation on tendon-bone healing. The study found that LIPUS stimulation twice a day was better for muscle and bone healing than the treatment once a day. Recently, (Xu et al., 2022b) observed in rats RC tear model that macrophage polarization may be a potential mechanism for LIPUS treatment of TBI repair. In conclusion, LIPUS therapy, as a non-invasive physical therapy, has been thoroughly studied in terms of promoting the mechanism of tendon-bone healing and the selection of treatment time and frequency. These results indicate that LIPUS therapy, as a technique to promote tendon-bone healing, may be widely used in clinic.

ESW is another potential non-invasive form of physical therapy that promotes tendon-bone healing. Chow et al. (2014) explored the effect of ESW on fibrocartilage regeneration during tendon-bone healing. It was found that ESW upregulated the expression of cell factors related to fibrocartilage production by providing mechanical signals, thus accelerating fibrocartilage regeneration at the healing interface. More studies are needed to confirm the efficacy and mechanism of ESW in promoting tendon-bone healing. In addition, intermittent negative pressure and Pulsed electromagnetic field (PEMF) therapy have been preliminarily explored to promote tenoon-bone healing after tendon repair with positive results (Sun et al., 2017; Huegel et al., 2022). More research is needed to confirm their specific efficacy and mechanism.

### 2.7 Combination therapy

In recent years, the physiological mechanism of tendon-bone healing has gradually become clear. Researchers are also trying to combine these methods to enhance their efficacy in promoting tendon-bone healing after tendon injury. Stem cells, cell factors, PRP and biomaterials were used in combination with each other, and positive effects were achieved (Table 7).

At present, stem cell and PRP therapy techniques are the mainstream methods to promote tendon-bone healing. The combined application of the two is also widely used in this field. Teng et al. (2016) studied the ability of PRP combined with BMSCs to promote tendon-bone healing. Studies have shown that PRP-BMSCs promote more mature healing of the tendon-bone interface, and the joint shows a higher functional load. Similarly, (Han et al., 2019a) explored the efficacy of combined MSCs and PRP in RC damage repair model. The results showed significantly enhanced signals related to angiogenesis, bone formation, and *in situ* tendon formation induced by combination therapy. The above studies confirm that the combination of MSCs and PRP can synergistically promote tendon-bone healing, which has great hope for promoting the treatment of tendon-bone healing. In addition, (McGoldrick et al., 2017) prepared extracellular matrix hydrogel loaded with PRP and ADSCs in order to make the drug release in the joint more lasting and better. It showed superior repair ability and biocompatibility in the rat model of Achilles tendon injury repair. Therefore, optimizing the effect of drug release based on the combination of stem cells and PRP may offer greater hope for promoting tendon-bone healing.

It was found that the combination of MSCs and demineralized bone matrix (DBM) can also enhance the role of MSCs in promoting tendon-bone healing. Thangarajah et al. (2016) explored the effect of DBM combined with MSCs on improving the function of the tendon-bone interface. In the treatment of patella tendon injury repair model, it was found that the combination effectively promoted the healing of the tendon-bone interface. Similarly, (Thangarajah et al., 2018) also studied the effect of the combination of DBM and MSCs on RCT healing. The results also showed that the combination of the two methods enhanced RC healing at an early stage and restored bone mineral density in the RC to pre-injury levels. These studies suggest that tendon repair using DBM and MSCs can achieve better tendon-bone healing early after surgery, and further studies are needed to determine its efficacy.

Furthermore, researchers have provided a new strategy for promoting tendon-bone healing. Combining biological scaffolds with tissue-specific stem cells may be a promising protocol for promoting tendon-bone healing. Hu et al. (2018) explored the synergistic therapeutic effect of collagen-filament scaffolds combined with derived stem cells on ACL regeneration. It was found that this treatment strategy significantly improved cartilage degeneration and reduced the severity of joint fibrosis. Similarly, (Chen et al., 2020) developed polylactic acid-glycolic acid (PLGA) scaffolds loaded with BMSCs. In a rabbit RCT repair model, it was found to promote tendon-bone healing by improving the biomechanical properties of the regenerated tendon. Besides, (Chen et al., 2019) studied the efficacy of scaffolds loaded with gene-edited BMSCs in improving RC repair. BMP-12 overexpressed rabbit BMSCs were used in this study. The results demonstrated that the PLGA scaffold loaded with BMSCs overexpressing BMP-12 could promote the healing of the tendon-bone interface, improve collagen tissue and increase fibrocartilage in rabbit RCT repair. In conclusion, biological scaffolds combined with MSCs are an effective strategy to improve tendon-bone healing, especially the combined use of MSCs that can be directed differentiation after gene modification.

## 3 Clinical study on promoting tendon-bone healing

As mentioned earlier, numerous strategies have been used in preclinical studies to promote tendon-bone healing, and some therapeutic strategies have also been used in clinical studies. Relevant clinical studies on stem cells and PRP have achieved certain curative effect, however, due to the lack of abundant clinical studies, some studies have produced negative curative effect (Table 8). Therefore, more clinical studies are needed to verify the safety and effectiveness of the above strategies, so as to accelerate their clinical application.

Currently, preclinical studies have shown that stem cell applications help promote tendon-bone healing after ACL reconstruction. However, the current clinical research results haven’t made a breakthrough. Alentorn-Geli et al. (2019) explored the clinical efficacy of ADRCs in the treatment of patients with ACL reconstruction. A matched cohort of 20 patients who underwent ADRCs infiltration followed by ACL reconstruction was compared with 19 patients who underwent the same surgery without ADRCs infiltration. The results found that patients treated with ADRCs at the time of ACL reconstruction had significantly improved knee function and graft maturity at 12 months. In addition, (Moon et al., 2021) studied whether allogeneic hUCB-MSCs could improve the clinical effect of human ACL reconstruction (KCT0000917). Allogeneic hUCB-MSCs were found to be safe for ACL reconstruction with no treatment-related adverse events at a 2-year follow-up. However, this study did not demonstrate a clinical advantage over allogeneic hUCB-MSCs. The application of stem cells to promote tendon-bone healing needs more clinical studies. After all, stem cells from different sources can differentiate in very different directions.

Studies of PRP in promoting tendon-bone healing have shown similar results to stem cells. Chen et al. (2022) retrospectively studied the effect of PRP on tendon-bone healing after ACL reconstruction. The results confirm that PRP can promote graft muscle and bone healing and improve knee function early. Recently, (Gong et al., 2022) studied the effect of PRP on tendon-bone healing and intraarticular graft maturation after ACL reconstruction (NCT04659447). In this study, it was found that PRP had no significant effect on reducing bone tunnel widening, accelerating tendon-bone healing, and improving knee joint function. However, this study have shown that PRP may promote the maturation of intraarticular grafts. In addition, (Pandey et al., 2016) performed PRP treatment on 52 RCT patients undergoing arthroscopic repair. The results showed that the application of moderately concentrated PRP could improve the clinical outcome of large cuff tear. PRP can also enhance the growth of blood vessels around the repair site early on. These studies suggest that PRP may promote tendon-bone healing at an early stage by promoting intraarticular graft maturation. However, more clinical studies are needed to confirm.

Preclinical studies have shown that Cap hybridization can promote fibrochondrogenesis of transplanted tendons. In the study (Mutsuzaki et al., 2019), eight patients underwent ACL reconstruction of quadriceps tendon transplantation by Cap hybridization. The patients were followed up for 2 months to 4 years. The results show that the Cap hybridization method is safe and feasible for ACL reconstruction in clinical trials, and can improve clinical outcomes. This study is only a one-arm clinical study, and its clinical efficacy needs to be verified by more controlled studies.

In addition, there are few clinical studies on physical therapy to promote tendon-bone healing. Wang et al. (2014) evaluated the influence of extracorporeal shock wave therapy (ESWT) on human ACL reconstruction. In this study, 26 patients underwent ESWT immediately after ACL reconstruction. Tibial tunnel radiographs in ESWT group were significantly lower than those in control group at 2 years after surgery (*p* = 0.018). The study results confirmed that ESWT significantly improved the subjective lysholm score of patients in the early stage after ACL reconstruction, and the tibial tunnel was significantly less than the control group in the long term. This study showed that ESWT after ACL reconstruction not only improved the clinical symptoms in the early stage, but also prevented the enlargement of the bone marrow tract in the long term. In the future, more clinical studies are needed to accelerate the pace of clinical application of this technology. Besides, a meta-analysis conducted by W. C. (Lai et al., 2021) evaluated the efficacy of LIPUS on tendon-to-bone healing. A total of 28 animal studies and two human studies met the inclusion criteria. Animal experiments demonstrated that LIPUS treatment significantly improved collagen content and organization, bone formation, fibrocartilage remodeling, and mechanical strength compared with control. Nevertheless, LIPUS for tendon injury disorders hasn’t improved clinical outcomes in human trials. The results of this study indicate that LIPUS is still not clinically effective in promoting tendine-bone healing. Therefore, more mechanism studies are needed to determine the mechanism by which physical therapy promotes tendine-bone healing, so as to regulate the selection of timing and parameters of physical therapy, so as to achieve better efficacy in promoting tendine-bone healing.

## 4 Discussion

It is well known that the major factor affecting recovery after ligament repair and reconstruction is tendon-bone healing (Mao et al., 2021). The main process of tendon-bone healing is the formation of fibrocartilage and mineralized bone (Zhao et al., 2022). As a result, numerous strategies have been developed to promote tendon-bone healing by promoting fibrochondral and bone formation pathways. This paper summarizes the current therapeutic measures used to promote tendon-bone healing, including biological materials, and compounds, stem cells, cell factors, and physical therapy. In addition, PRP and exosomes have been developed on the basis of stem cell and cytokine therapy. At present, most of the above research methods have achieved gratifying results in preclinical studies. However, due to the relatively few clinical studies, most of the above strategies have not been widely carried out in clinical practice.

MSCs transplantation has been widely used in tendon and bone repair, and has shown good efficacy in promoting tendon-bone healing (Jang et al., 2015; Xu et al., 2019; Chen et al., 2021). Different MSCs from different sources have different differentiation directions, which leads to different MSCs play different roles in promoting tendon-bone healing. Therefore, tissue-derived MSCs such as synovium and adipose tissue are being developed for application in tendon-bone healing (Kosaka et al., 2016; Matsumoto et al., 2021). Researchers have also attempted to combine MSCs and PRP for tendon-bone healing, and achieved good results (Teng et al., 2016; Han et al., 2019a). In recent years, researchers have found that MSCs exercise their biological regulatory function by secreting exosomes. A large number of studies have been conducted on the application of MSCs-Exos to promote tendon-bone healing, and preliminary results have been gratifying (Shi et al., 2020; Ren et al., 2021; Han et al., 2022; Li et al., 2022). In addition, with the development of genetic engineering technology, researchers have also begun to modify MSCs through gene modification methods, such as BMP2 and bFGF (76), so that they can target differentiation and promote tendon-bone healing. Therefore, genetically modified stem cell techniques and stem-cell derived exosomes may be promising strategies for promoting tendine-bone healing in the future. PRP contains abundant cell factors that can promote tendon and bone tissue repair, so it is widely used in tendon, bone tissue and wound repair (Figueroa et al., 2015). PRP has been shown in basic studies to be beneficial to tendon-bone interface healing early by promoting fibro chondral formation. However, PRP hasn’t achieved satisfactory efficacy in clinical studies. Recently, Zhu et al. (2022b) conducted a systematic review and meta-analysis to explore the clinical efficacy of PRP in ACL reconstruction. A total of 14 Chinese and English studies were included in this study. The results showed that PRP in ACL reconstruction can reduce postoperative pain and improve knee function in the short to medium term, but not in the long term. The study also found that PRP didn’t improve knee stability or tunnel enlargement, nor did it accelerate graft healing. There are also limitations in this study. The injection volume, concentration, intensity and injection times of PRP in the study were different in different studies. Secondly, the source of the transplanted tendon during ACL reconstruction is also different. Therefore, more studies are needed in the future to further verify the role of PRP in promoting tendine-bone healing on the one hand, and determine the method and specific dosage of PRP on the other hand, so as to facilitate its widespread clinical promotion. RCT repair and ACL reconstruction surgery determined that local administration was the preferred option (Saab et al., 2022). However, local application of the drug may result in short joint retention time or incongruous onset of the drug and the physiological stages of tendon-bone healing (Zhao et al., 2022). Hydrogel microspheres have the advantages of minimally invasive, prolonged drug retention time and high drug loading efficiency, and have become a promising drug carrier in the biological field. Currently, it has been used to promote tendon-bone healing as a drug delivery tool and has shown good function in joint local administration (Zhao et al., 2022). It is believed that with the rapid development of biomaterial technology, hydrogel microspheres may be the most effective drug carrier to promote tendon-bone healing.

Physical therapy is widely used in sports medicine because of its non-trauma. It also shows great potential in promoting tendon-bone healing (Chow et al., 2014; Lu et al., 2016b). The study of Wang et al. (2014) confirmed that ESWT could avoid the enlargement of bone marrow tract in the long term after ACL reconstruction. As mentioned above, most of the previous strategies mainly act on the early stage of tendon-bone healing, and most of the long-term curative effects aren’t obvious. Therefore, physical therapy in combination with other therapies that promote tendon-bone healing may compensate for the long-term lack of efficacy of these methods.

## 5 Conclusion

Therefore, tendon-bone healing determines the outcome of RCT repair and ACL reconstruction. As a result, researchers have tried a variety of strategies to promote tendon-bone healing. At present, biomaterials and compounds, stem cells, cell factors, PRP, exosomes, physical therapy and other technologies have been widely used in the study of promoting tendon-bone healing, and have achieved good efficacy. With the gradual maturation of these techniques and further study of the mechanism of tendon-bone healing, the strategies to promote tendon-bone healing will certainly make breakthroughs.
